# Molecular prevalence of *Coxiella burnetii* in cheese samples: Systematic review and meta‐analysis

**DOI:** 10.1002/vms3.1335

**Published:** 2023-12-15

**Authors:** Berna Yanmaz, Ediz Kagan Ozgen

**Affiliations:** ^1^ Department of Public Health Faculty of Veterinary Medicine Burdur Mehmet Akif Ersoy University Burdur Turkey; ^2^ Department of Microbiology Faculty of Veterinary Medicine Atatürk University Erzurum Turkey

**Keywords:** coxiellosis, PCR, real‐time, Q fever, zoonosis

## Abstract

**Background:**

Cheese is a popular dairy product consumed worldwide, and it has been implicated as a source of *Coxiella burnetii* infections.

**Objectives:**

The present study aimed to describe the molecular prevalence and source analysis of *C. burnetii* in cheese samples.

**Methods:**

A systematic literature search was conducted using the Medline/PubMed, Science Direct, Web of Science, Scopus, and Google Scholar databases to identify studies reporting the molecular prevalence of *C. burnetii* in cheese samples. The pooled prevalence of *C. burnetii* in cheese samples was estimated using a random‐effects model.

**Results:**

A meta‐analysis was conducted using the mean and standard deviation values obtained from 13 original studies. The overall molecular prevalence of *C. burnetii* in cheese was estimated to be 25.2% (95% confidence interval [CI]: 13.1%–39.7%). The *I* ^2^ value of 96.3% (CI_95%_ 94.9–97.3) suggested high heterogeneity, with a *τ* ^2^ of 0.642 (CI_95%_ −0.141 to 0.881), and an *χ* ^2^ statistic of 323.77 (*p* < 0.0001).

**Conclusions:**

In conclusion, our meta‐analysis provides a thorough assessment of the molecular prevalence and source analysis of *C. burnetii* in cheese samples.

## Graphical abstract

The overall molecular prevalence of *Coxiella burnetii* in cheese was estimated to be 25.2%.

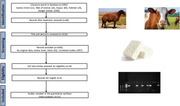

## 1 INTRODUCTION

*Coxiella burnetii* is an obligate intracellular bacterium that causes the zoonotic disease *Q fever*, which can affect both humans and animals (Ozgen et al., 2022). The transmission of *C. burnetii* to humans can occur through inhalation of contaminated dust or contact with infected animals, particularly livestock (Tan et al., 2022). However, it has been reported that ingestion of contaminated food, especially unpasteurized milk and dairy products, can also be a source of human infection (Eldin et al., 2017).

Cheese is a popular dairy product consumed worldwide, and it has been implicated as a source of *C. burnetii* infections (Gale et al., 2015). Therefore, it is important to determine the molecular prevalence of *C. burnetii* in cheese to assess the potential risk of transmission to humans. Molecular detection methods, such as PCR and qPCR, have been increasingly used to detect *C. burnetii* in various dairy products (Basanisi et al., 2022; Petruzzelli et al., 2013).

Meta‐analysis is a statistical technique used to combine and analyse data from multiple independent studies and has become increasingly popular in the field of food science and nutrition (Atamaleki et al., 2019). One scientific reason for using meta‐analysis in food‐related studies is that it allows researchers to gain a more precise estimate of the effect of a particular food or dietary pattern on health outcomes (Hamidiyan et al., 2018). By pooling data from multiple studies, meta‐analysis can increase the statistical power of the analysis and reduce the impact of random variation in individual studies, thereby providing a more accurate estimate of the true effect size (Sutton et al., 2001). Furthermore, meta‐analysis can also help identify sources of heterogeneity or inconsistency across studies and can be used to explore potential sources of bias or confounding factors. Thus, meta‐analysis can provide a more robust and comprehensive evaluation of the evidence base in food‐related studies and can help guide future research and public health policy (Khaneghah et al., 2018; Khaneghah et al., 2019).

In this study, a systematic review and meta‐analysis were conducted to determine the molecular prevalence and source analysis of *C. burnetii* in cheese samples. The findings of this study could provide important insights into the potential public health risks associated with the consumption of contaminated cheese and inform effective preventive measures to reduce the risk of transmission of *C. burnetii* to humans.

## 2 MATERIALS AND METHODS

### 2.1 Literature search and study selection

The systematic review and meta‐analysis followed the Preferred Reporting Items for Systematic Reviews and Meta‐Analyses guidelines (Moher et al., 2009). A systematic literature search was conducted using the Medline/PubMed, Science Direct, Web of Science, Scopus, and Google Scholar databases to identify studies reporting the molecular prevalence of *C. burnetii* in cheese samples. The search was conducted using the following keywords: (‘*C. burnetii’* or ‘*Q fever’* or ‘*C. burnetii’* or ‘coxiellosis’) and (‘cheese’) and (‘molecular’ or ‘PCR’ or ‘real‐time PCR’ or ‘qPCR’ or ‘conventional PCR’ or ‘nested PCR’ or ‘molecular diagnosis’ or ‘polymerase chain reaction’). The search was limited to studies published in English between January 2000 and April 2023.

### 2.2 Inclusion criteria

To identify any additional relevant studies, the reference lists of the identified studies were searched by hand. Full texts of potentially eligible studies were reviewed to determine if they met the inclusion criteria. Studies were considered eligible for inclusion if they met the following criteria: (1) reported the molecular prevalence of *C. burnetii* in cheese samples, (2) used molecular methods to detect the presence of *C. burnetii* DNA in cheese samples, (3) provided sufficient data to calculate the prevalence estimate and its confidence interval (CI) and (4) were primary studies, not reviews.

### 2.3 Data extraction and meta‐analysis

A standardized form was used to extract data from eligible studies. This form included the following information: author, year of publication, country, study area, period of study, origin of cheese, milk species, kind of production, country classification, DNA extraction, molecular approach, target gene, number of cheese samples tested, number of positive samples, prevalence and CI.

The meta‐analysis included in this study used the *C. burnetii* determined by molecular methods in cheese samples as the dependent variable, which served as the effect size for the analysis. To perform the meta‐analysis of proportions, the previous methodology was followed (Wang, 2018). Heterogeneity among studies was initially assessed using Cochran's *Q* (*χ* ^2^) test, which examines the null hypothesis of homogeneity. Subsequently, the extent of heterogeneity was quantified using Higgins’ *I* ^2^ statistic (Borenstein et al., 2011). The level of heterogeneity was then measured to select the appropriate model for estimating the overall weighted *C. burnetii* prevalence. Given that the level of heterogeneity was high, a random‐effects model was used to account for both within‐study variance (sampling error) and between‐studies variance (*τ* ^2^).

Potential origins of heterogeneity were explored through the examination of moderating factors. Five distinct moderators were subjected to evaluation: (1) distinction between developed and developing countries, (2) milk source (goat or sheep in comparison to cow), (3) target gene (the transposon‐like repetitive region of the bacterial genome (IS1111) as opposed to others), (4) extraction method (commercial vs. non‐commercial) and (5) methodological approach used in DNA extraction (mechanical vs. non‐mechanical). A subgroup analysis was performed for the categorical moderators, which were analysed using a mixed‐effects model. The statistical significance of the moderators was evaluated by an omnibus test (QM) within the mixed‐effects model (Viechtbauer 2010). The *R* ^2^ index was used to explore the proportion of heterogeneity accounted for by each moderator. To further explore heterogeneity among the studies, meta‐regression was utilized. All moderators and their interactions were entered into the initial model, and non‐significant terms were then dropped stepwise (from lowest *R* ^2^ to highest *R* ^2^) (Li et al., 2020). The association among moderators (country classification, milk source and methodological approach employed) was assessed by the Pearson correlation coefficient (*r*). Results from the meta‐analysis, along with the corresponding 95% CIs, were summarized using forest plots. Egger's test was used to test for the possibility of publication bias for studies with low or high effect sizes (Egger et al., 1997). All assessments were conducted using commercial software (Med‐Calc; version 20.110; MedCalc Software Ltd).

## 3 RESULTS

**FIGURE 1:**
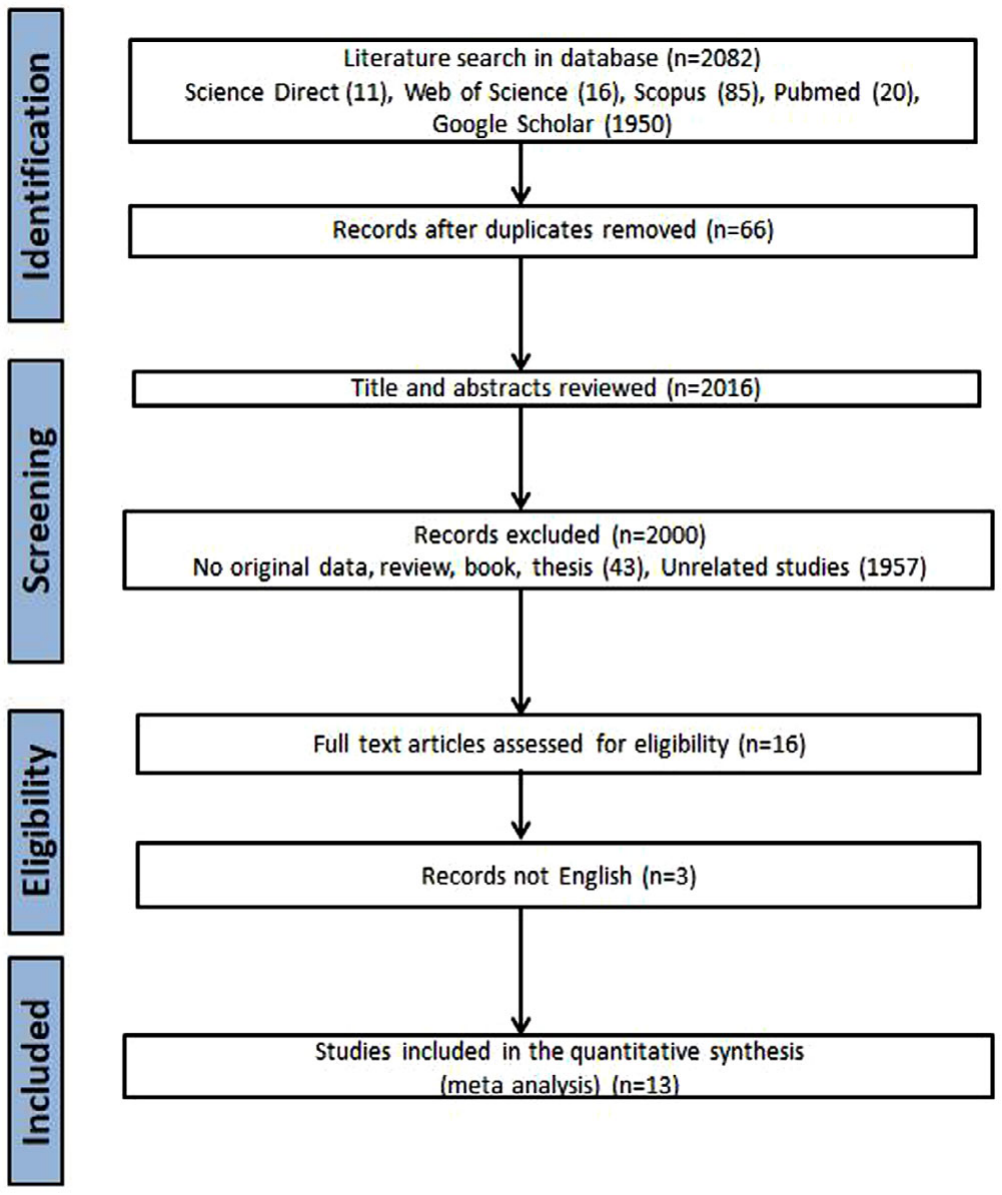
Preferred Reporting Items for Systematic Reviews and Meta‐Analyses (PRISMA) flow diagram illustrating the study selection process for the systematic review and meta‐analysis of the molecular prevalence and source analysis of *Coxiella burnetii* in cheese samples.

An initial search yielded a total of 2082 articles, out of which 13 met the inclusion criteria. The study selection process is summarized in Figure 1, and the characteristics of the included studies are presented in Table 1. These studies were conducted in eight different countries (Japan, Italy [3 studies], Poland, France, Iran [3 studies], Spain, Brazil [2 studies], Latvia) and were published between 2011 and 2022. A meta‐analysis was conducted using the mean and standard deviation values obtained from 13 original studies. The sample sizes ranged from 28 to 178 cheese samples, collected over 15 years (2005 to 2020). The studies employed real‐time PCR (*n* = 8), nested PCR (*n* = 3), touch‐down PCR (*n* = 1) and droplet digital PCR (*n* = 1). The *IS1111* was the gene most frequently targeted in these PCRs (*n* = 9), followed by *com1* (*n* = 2), *icd* (*n* = 1), *IS30A* (*n* = 1) and *htp‐B* (*n* = 1). Seven studies were conducted in developed countries and six in developing countries. Regarding DNA extraction methods, 11 studies utilized commercial extraction protocols, whereas two trials employed non‐commercial methodologies. Nucleic acid isolation from food samples was accomplished using mechanical procedures in nine studies, whereas four studies abstained from incorporating mechanical procedures for nucleic acid isolation (Table 1).

**TABLE 1:** Characteristics and main results of the eligible studies ordered by molecular prevalence of *Coxiella burnetii* in cheese samples.

Author and Year	Country	Study area	Period of study	Origin of cheese	Milk species	Kind of Production	Country classification	DNA extraction			Molecular approach	Target gene	Cheese samples tested	Positive cheese samples	Prevalence	95% CI
								MP	EP	Method						
Nascimento et al. (2021)	Brazil	Cerrado Mineiro	2020	Brazil	Cow	Artisanal	Developing	+	+	CK	qPCR	*IS1111*	87	4	4.6	1.1–10.3
Abdali et al. (2018)	Iran	Shiraz	2016	ND	ND	ND	Developing	−	+	CK	nPCR	*com1*	46	2	4.3	0.5–14.8
Khanzadi et al. (2014)	Iran	Mashhad	2012	Iran	Sheep	ND	Developing	+	+	CK	TdPCR	*IS1111*	28	2	7.1	0.8–23.5
Mosleh et al. (2018)	Iran	Tehran	2018	Iran	ND	Artisanal	Developing	+	+	NCK	qPCR	*IS1111*	40	3	7.5	1.5–20.3
Basanisi et al. (2022)	Italy	Apulia and Basilicata	2018–2020	Italy	Sheep and goat	ND	Developed	+	+	CK	DdPCR	*IS1111, icd*	128	10	7.8	3.8–13.8
Rozental et al. (2020)	Brazil	Serro microregion	2020	Brazil	Cow	Artisanal	Developing	+	+	CK	nPCR	*IS1111*	53	5	9.4	3.1–20.6
Hirai et al. (2012)	Japan	Tokyo	2005–2008	12^a^ country	ND	ND	Developed	+	+	NCK	nPCR	*com1/htp‐B/icd*	147	28	19.0	13.0–26.3
Capuano et al. (2012)	Italy	Southern area	2010–2011	Italy	Cow, buffalo, sheep and goat	Artisanal/Commercial	Developed	+	+	CK	qPCR	*IS1111*	169	36	21.3	15.3–28.2
Barandika et al. (2019)	Spain	Spain	2019	Spanish	Sheep	Artisanal	Developed	+	+	CK	qPCR	*IS1111*	67	20	29.9	19.2–42.2
Szymańska‐Czerwińska et al. (2019)	Poland	Poland	2014–2017	Polish	ND	ND	Developing	‐	+	CK	qPCR	*IS1111*	40	34	85.0	70.2–94.3
Galiero et al. (2016)	Italy	Tuscany	2014–2015	Italy	Sheep, cow and goat	Artisanal/Commercial	Developed	+	+	CK	qPCR	*IS1111*	84	27	32.1	27.7–49.3
Valkovska et al. (2021)	Latvia	Riga	ND	Latvia	Cow and goat	ND	Developed	−	+	CK	qPCR	*IS1111*	40	24	60.0	43.3–75.1
Eldin et al. (2013)	France	Alps and Provence	ND	ND	Cow, sheep and goat	Artisanal/Commercial	Developed	−	+	CK	qPCR	*IS1111, IS30A*	178	117	65.7	58.2–72.6

Abbreviations: CI, confidence interval; CK: commercial kit; DdPCR: droplet digital PCR; EP: enzymatic processes; NCK: non‐commercial kit; ND: Not determined; nPCR: nested PCR; MP: mechanical procedure; TdPCR: touch‐down PCR; qPCR: real time PCR.

^a^France, Japan, Italy, Switzerland, Denmark, Australia, The Netherlands, New Zealand, Germany, Cyprus, Greece and Spain.

**FIGURE 2:**
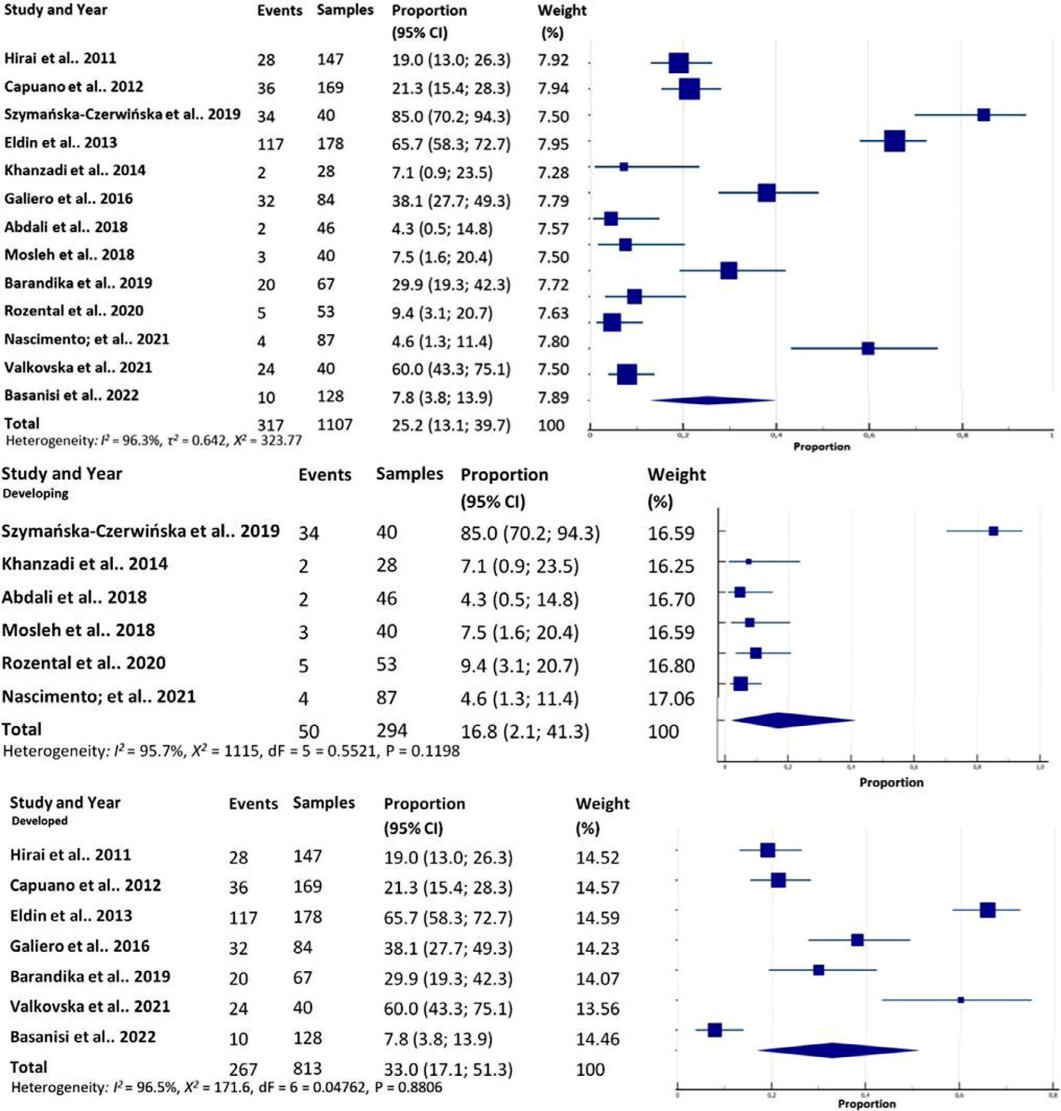
Forest plot illustrating meta‐analysis of *Coxiella burnetii* prevalence in cheese samples from thirteen studies conforming to inclusion criteria in the systematic review. Subgroup analysis of *C. burnetii* prevalence in cheese samples differentiating developed and developing countries.

The median size of eligible studies was 67 cheese samples, and out of the total 1107 cheese samples, 317 were diagnosed as positive by molecular techniques. The percentage of positive cheese samples across the studies ranged from 4.3% to 85%. The overall molecular prevalence of *C. burnetii* in cheese was estimated to be 25.2% (95% CI: 13.1%–39.7%). The *I* ^2^ value of 96.3% (CI_95%_ 94.9–97.3) suggested high heterogeneity, with a *τ* ^2^ of 0.642 (CI_95%_ −0.141 to 0.881), and an *χ* ^2^ statistic of 323.77 (*p* < 0.0001). The overall meta‐analysis is shown in a forest plot (Figure 2). The meta‐analysis showed no significant evidence of publication bias based on Egger's test (*p* = 0.715).

**FIGURE 3:**
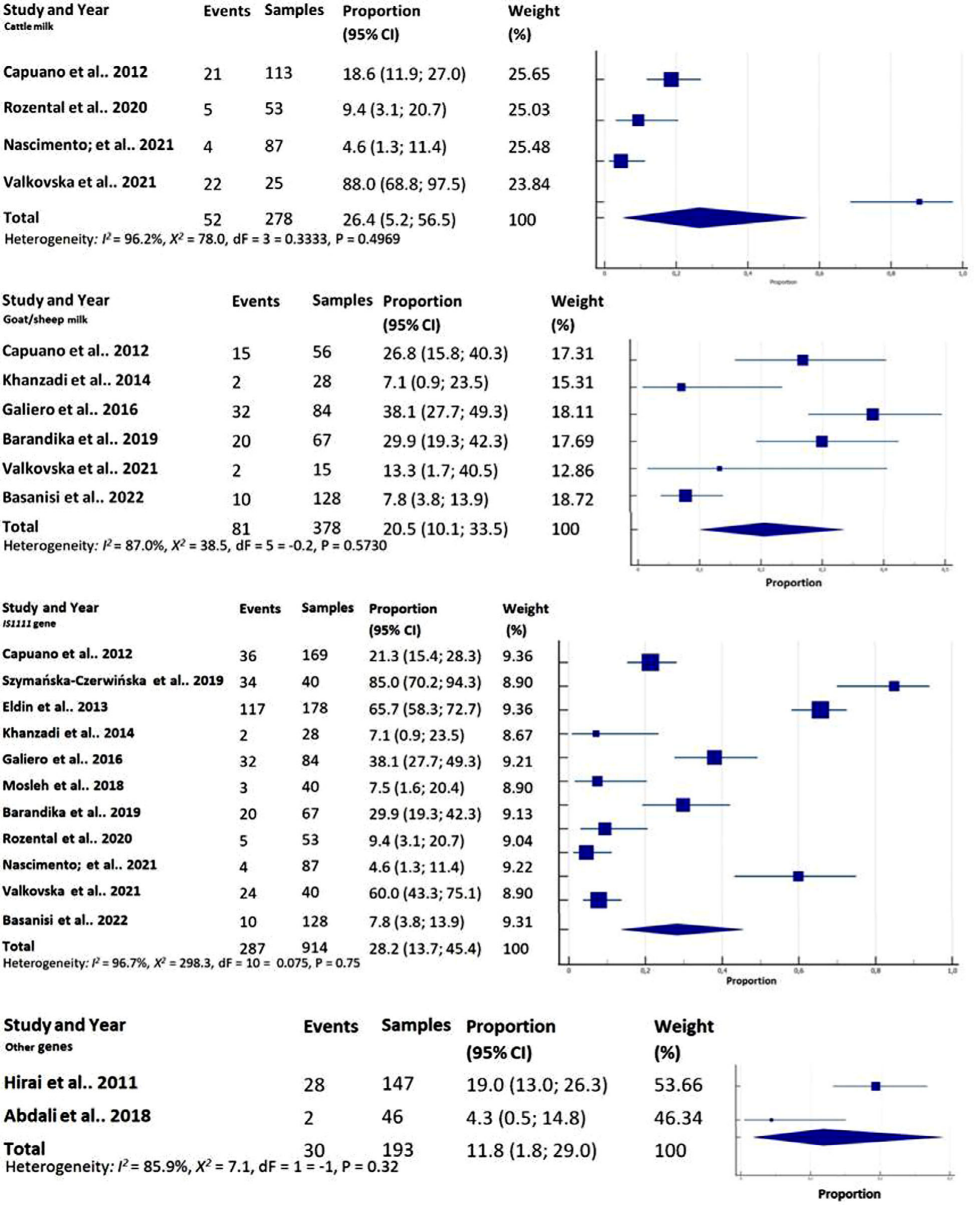
Subgroup analysis of *Coxiella burnetii* prevalence in cheese samples based on milk *source*: cattle milk vs. goat or sheep milk. Subgroup analysis of *C. burnetii* prevalence in cheese samples comparing *IS1111* gene detection with other genes.

The weighted average prevalence of *C. burnetii* in developed countries was 33.0% (CI_95%_ 17.1–51.3) (*I* ^2^ = 96.5%; *χ* ^2^ = 171.6, *p* < 0.0001; QM (dF = 6 = 0.04762, *p* = 0.8806), whereas in developing countries, it was 16.8% (CI_95%_ 2.1–41.3), *I* ^2^ = 95.7%; *χ* ^2^ = 115.0, *p* < 0.0001; QM (dF = 5 = 0.5521, *p* = 0.1198) (Figure 2). Cheese samples from cattle milk had a prevalence 26.4% (CI_95%_ 5.2–56.5), *I* ^2^ = 96.2%; *χ* ^2^ = 78.0, *p* < 0.0001; QM (dF = 3 = 0.3333, *p* = 0.4969), whereas in goat or sheep milk, the prevalence was 20.5% (CI_95%_ 10.1–33.5), *I* ^2^ = 87.0%; *χ* ^2^ = 38.5, *p* < 0.0001; QM (dF = 5 = −0.2, *p* = 0.5730) (Figure 3).

**FIGURE 4:**
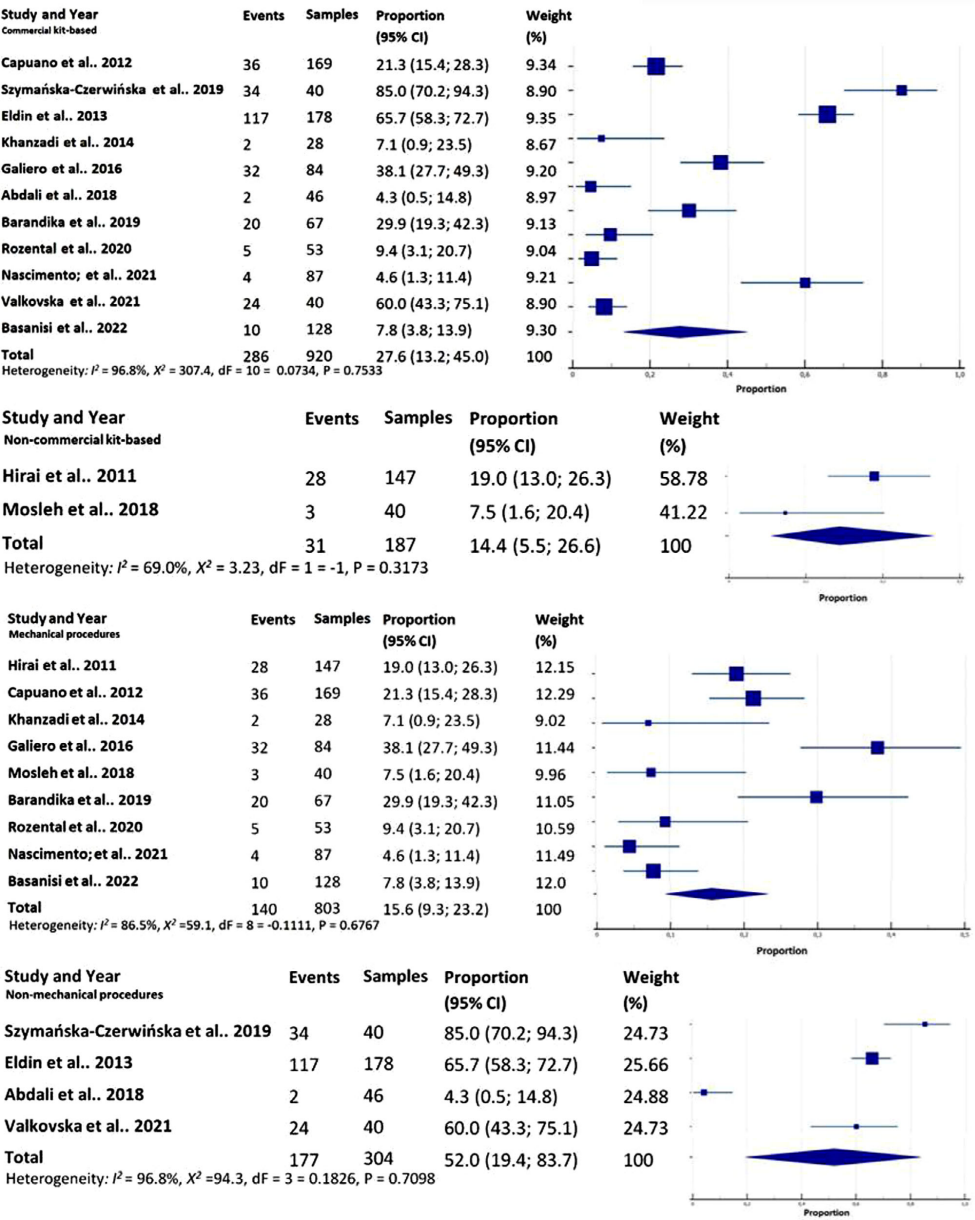
Subgroup analysis of *Coxiella burnetii* prevalence in cheese samples commercial kit‐based vs. non‐commercial kit‐based methods. Subgroup analysis of *C. burnetii* prevalence in cheese samples comparing mechanical procedures with non‐mechanical procedures for sample processing.

In the context of the subgroup analysis comparing the target gene, the observed prevalence was 28.2% (CI_95%_ 13.7–45.4), *I* ^2^ = 96.7%; *X* ^2^ = 298.3, *p* < 0.0001; QM (dF = 10 = 0.075, *p* = 0.75) for the *IS1111* gene. In contrast, the prevalence for other genes was found to be 11.8% (CI_95%_ 1.8–29.0), *I* ^2^ = 85.9%; *X* ^2^ = 7.1, *p* = 0.0078; QM (dF = 1 = −1, *p* = 0.32) (Figure 3). Turning to the methodologies employed, the prevalence derived from commercial kit‐based methods was 27.6% (CI_95%_ 13.2–45.0), *I* ^2^ = 96.8%; *χ* ^2^ = 307.4, *p* < 0.0001; QM (dF = 10 = 0.0734, *p* = 0.7533). In contrast, non‐commercial kit‐based methods yielded a prevalence of 14.4% (CI_95%_ 5.5–26.6), *I* ^2^ = 69.0%; *χ* ^2^ = 3.23, *p* = 0.0725; QM (dF = 1 = −1, *p* = 0.3173) (Figure 4). Shifting the focus to sample processing procedures, mechanical approaches yielded a prevalence of 15.6% (CI_95%_ 9.3–23.2), *I* ^2^ = 86.5%; *χ* ^2^ = 59.1, *p* < 0.0001; QM (dF = 8 = −0.1111, *p* = 0.6767). Conversely, non‐mechanical procedures resulted in a prevalence of 52.0% (CI_95%_ 19.4–83.7), *I* ^2^ = 96.8%; *χ* ^2^ = 94.3, *p* < 0.0001; QM (dF = 3 = 0.1826, *p* = 0.7098) (Figure 4). No significant correlation was identified between the mechanical and non‐mechanical approaches (*r* = 0.690, *p* = 0.309). There was no substantial correlation observed between milk type and the source of the studies (*r* = −0.011, *p* = 0.989). Moreover, no significant correlation was evident between developing and developed countries (*r* = −0.255, *p* = 0.626).

## 4 DISCUSSION

The global consumption of cheese and cheese‐related products is increasing, with a wide variety of cheese types consumed across different cultures and regions. Europe is the largest consumer of cheese, accounting for over 40% of the global cheese consumption. The consumption of cheese is deeply ingrained in European culture, with each country having its unique cheese‐making traditions and varieties. The United States is the second‐largest consumer of cheese globally, with an average annual consumption of over 37 pounds of cheese per person (Khanal et al., 2019). The consumption of raw milk cheese is common in some regions, particularly in Europe, where it is considered a traditional food. Raw milk cheese is made from unpasteurized milk, which has not undergone the process of heating to kill bacteria (Fusco et al., 2020). Several studies have investigated the prevalence of *C. burnetii* in raw milk and cheese, and the results have shown that the bacteria can be present in both products (Abdali et al., 2018; Barandika et al., 2019; Basanisi et al., 2022). The global molecular prevalence of *C. burnetii* in milk samples has been estimated by meta‐analysis (Rabaza et al., 2021). We carried out a comprehensive systematic review of the literature using keyword‐based searches to determine the global molecular prevalence of *C. burnetii* in cheese samples. Data from cross‐sectional studies that met our inclusion criteria were extracted and included in a meta‐analysis.

Our systematic review and meta‐analysis on the molecular prevalence of *C. burnetii* in cheese samples provide valuable insights into the global distribution and prevalence of this bacterium in cheese products. The estimated overall molecular prevalence of *C. burnetii* in cheese was found to be 25.2%, with a 95% CI of 13.1%–39.7%. These findings are consistent with previous studies that have reported the presence of *C. burnetii* in cheese samples from various regions worldwide (Basanisi et al., 2022; Eldin et al., 2013; Gyuranecz et al., 2012). The high heterogeneity observed across the studies, as indicated by the *I* ^2^ value of 96.3%, highlights the need for caution in interpreting these results. The variation in prevalence estimates across different regions and types of cheese may be attributed to differences in sampling methods, sample sizes and the sensitivity and specificity of the molecular techniques used for diagnosis. Additionally, differences in the prevalence of *C. burnetii* in animal populations may also contribute to the observed heterogeneity (Pearson et al., 2014). Despite the high heterogeneity, the lack of significant evidence of publication bias based on Egger's test suggests that the results are reliable and representative of the available literature.

Frequently encountered in the isolation of nucleic acids from food samples are four distinct processes: (1) mechanical procedures, including homogenization and filtration; (2) enzymatic processes; (3) DNA extraction; and (4) DNA purification (Jana et al., 2020). Due to the uneven distribution of microorganisms within food matrices, homogenization plays a crucial role in enhancing the diagnostic efficacy (Jana et al., 2020). The aggregated positivity rate observed in studies that undertook DNA extraction without mechanical treatment was comparatively elevated in comparison to those studies that incorporated mechanical processing during the DNA extraction procedure. Enzymatic processes was administered in all studies incorporated within the investigation. Concerning DNA extraction methods, 84.6% of trials employed commercial extraction protocols, whereas two trials conducted in Iran and Japan employed non‐commercial approaches. Among the studies employing non‐commercial methods for DNA extraction, a lower positivity rate is evident compared to the studies utilizing commercial kits. It is noteworthy that milk and dairy products may contain constituents such as fat and protein, which have the potential to impede PCR‐based assays (Baptista et al., 2021; Jana et al., 2020). Notably, extraction methodologies based on spin columns have demonstrated effectiveness in mitigating inhibitory substances and facilitating the retrieval of abundant DNA from milk and dairy products (Baptista et al., 2021).

However, 84.6% (11/13) of the studies included in this research employed the diagnostic method targeting the *IS1111* gene region. However, in one study, the *com1* gene region was utilized, whereas another study employed *com1*, *htp‐B*, and *icd* gene regions. In studies involving the *IS1111* gene region, the ratio of positive samples to the total number of cheese samples was 29.2%. However, in the two additional studies conducted in Iran and Japan, this rate dropped to 15.5%. The *IS1111* gene region exhibits varying copy numbers depending on the strain, resulting in enhanced sensitivity (Rabaza et al., 2021; WOAH, 2018). The elevated positivity rate observed in studies focusing on the *IS1111* gene region is believed to stem from its heightened sensitivity.

In the molecular investigations, diverse methodologies have been employed in prior studies, encompassing qPCR (*n* = 8), nPCR (*n* = 3), TdPCR (*n* = 1) and DdPCR (*n* = 1). These approaches yielded total positivity rates of 35.1%, 14.2%, 7.1% and 7.8%, respectively. The qPCR stands as a robust methodology in comparison to other PCR‐based assays, as it facilitates the acquisition of quantitative data regarding the quantity of nucleic acid within the scrutinized specimen (Taylor et al., 2019). In the studies under consideration within this study, the diagnosis of *C. burnetii* involved the targeting of the *IS1111* gene region using qPCR. However, owing to the copy number variability across strains, the *IS1111* gene region cannot be precisely quantified through the employed targeted qPCR methodology (WOAH, 2018). Comparatively, nested PCR aimed at the *com1* gene region exhibits lower sensitivity when contrasted with touchdown PCR that targets the *IS1111* gene region (Kargar et al., 2015). Within the showcased studies, the prevalence of qPCR and the *IS1111* gene region targeting is notable across various PCR techniques. The incorporation of these dual strategies in the diagnosis of *C. burnetii* is poised to deliver more accurate outcomes.

The present study provides important findings on the prevalence of *C. burnetii* in cheese samples across different countries and types of milk. Interestingly, the results show a significantly higher prevalence of *C. burnetii* in developed countries (33.0%) compared to developing countries (16.8%). This difference in prevalence may be attributed to differences in the level of awareness, hygiene practices and animal husbandry practices between developed and developing countries (Mura et al., 2014). The study also found a higher prevalence of *C. burnetii* in cheese samples obtained from cattle milk (26.4%) compared to those obtained from goat or sheep milk (20.5%). This is in contrast with previous studies that have reported a higher prevalence of *C. burnetii* in small ruminants than in cattle (Arricau‐Bouvery et al., 2005; Guatteo et al., 2011). This is possibly because infected cattle can shed viable C. burnetii in milk for longer than one year (Enright et al., 1957). It should be noted that the prevalence rates reported in this study should be interpreted with caution due to the high degree of heterogeneity observed in the data (*I* ^2^ > 80%). This heterogeneity may be due to variations in the sampling methods, testing procedures and diagnostic criteria used in different studies. Therefore, future studies should aim to standardize these variables to obtain more reliable prevalence estimates.

The presence of *C. burnetii* in cheese products poses a potential risk to human health, as this bacterium is a known zoonotic pathogen that can cause Q fever in humans. The risk of transmission of *C. burnetii* through contaminated cheese products can be minimized through appropriate measures, including the use of pasteurized milk in cheese production, proper sanitation and hygiene practices and appropriate labelling and packaging of cheese products (Owusu‐Kwarteng et al., 2020). There is a need for greater awareness of the potential risks associated with the consumption of raw milk cheese and the transmission of *C. burnetii*. Producers and consumers should take appropriate measures to minimize the risk of contamination, including the use of pasteurized milk in cheese production, proper sanitation and hygiene practices and appropriate labelling and packaging of cheese products.

The findings of this study highlight the importance of implementing effective preventive measures to reduce the risk of transmission of *C. burnetii* to humans through contaminated cheese. These measures may include the use of pasteurized milk, strict hygiene measures during cheese production and consumer education on the potential risks of consuming unpasteurized cheese. However, our meta‐analysis has some limitations that need to be taken into consideration when interpreting the results. First, the studies included in our analysis were conducted over a period of several years, and there may be variations in the prevalence of *C. burnetii* over time due to changes in animal husbandry practices, weather conditions and other factors. Second, the number of included studies in developed countries was higher than developing countries, which may limit the generalizability of the findings to other regions. Third, the molecular methods used to detect *C. burnetii* in cheese samples varied among the included studies, which may have contributed to the observed heterogeneity. Finally, publication bias cannot be ruled out, as studies with negative results may not have been published.

## 5 CONCLUSIONS

Our meta‐analysis presents a comprehensive estimation of the molecular prevalence and source analysis of *C. burnetii* in cheese samples. The substantial heterogeneity observed underscores the necessity for standardized sampling and PCR protocols to ensure cross‐study comparability. By doing so, identification of factors contributing to *C. burnetii* contamination in cheese becomes feasible, facilitating effective measures to prevent Q fever transmission to humans. Furthermore, our study reveals discernible prevalence patterns linked to distinct genetic markers, methodologies and sample processing procedures within the context of *C. burnetii* prevalence in cheese. The prominent prevalence observed for the *IS1111* gene underscores its substantive presence, whereas other genes display comparatively lower prevalence rates. Methodological variations, particularly between commercial and non‐commercial kit‐based methods, yield disparate prevalence outcomes. Variations in sample processing procedures similarly yield significant differences in prevalence rates between mechanical and non‐mechanical techniques.

Our findings highlight the absence of substantial correlations among milk type, study origin and development status with *C. burnetii* prevalence. This underscores the complex and multifaceted character of these relationships. This study further underscores the imperative of improving hygiene and animal husbandry practices in both developed and developing nations to effectively lower the potential for *C. burnetii* contamination in cheese products.

## AUTHOR CONTRIBUTIONS

*Conceptualization; formal analysis; investigation; methodology; visualization; writing – original draft*: Berna Yanmaz. *Conceptualization; investigation; methodology; writing – review and editing*: Ediz Kagan Ozgen.

## CONFLICT OF INTEREST STATEMENT

The authors declare no conflicts of interest.

## FUNDING INFORMATION

This research received no specific grant from any funding agency in the public, commercial or not‐for‐profit sectors.

## ETHICS STATEMENT

No ethical approval was required as this study does not involve any animal subjects.

### PEER REVIEW

The peer review history for this article is available at https://www.webofscience.com/api/gateway/wos/peer‐review/10.1002/vms3.1335.

## Data Availability

The data that support the findings of this study are available from the corresponding author upon reasonable request.

## References

[vms31335-bib-0001] Abdali, F. , Hosseinzadeh, S. , Berizi, E. , & Shams, S. (2018). Prevalence of *Coxiella burnetii* in unpasteurized dairy products using nested PCR assay. Iranian Journal of Microbiology, 10(4), 220. 30483373 PMC6243153

[vms31335-bib-0002] Arricau‐Bouvery, N. , Souriau, A. , Bodier, C. C. , Dufour, P. , & Rousset, E. (2005). *Coxiella burnetii* shedding by dairy cows. Veterinary Research, 36(6), 827–834.16120256

[vms31335-bib-0003] Atamaleki, A. , Yazdanbakhsh, A. , Fakhri, Y. , Mahdipour, F. , Khodakarim, S. , & Khaneghah, A. M. (2019). The concentration of potentially toxic elements (PTEs) in the onion and tomato irrigated by wastewater: A systematic review; meta‐analysis and health risk assessment. Food Research International, 125, 108518. 31554079 10.1016/j.foodres.2019.108518

[vms31335-bib-0004] Baptista, M. , Cunha, J. T. , & Domingues, L. (2021). DNA‐based approaches for dairy products authentication: A review and perspectives. Trends in Food Science & Technology, 109, 386–397.

[vms31335-bib-0005] Barandika, J. F. , Alvarez‐Alonso, R. , Jado, I. , Hurtado, A. , & García‐Pérez, A. L. (2019). Viable *Coxiella burnetii* in hard cheeses made with unpasteurized milk. International Journal of Food Microbiology, 303, 42–45. 31132730 10.1016/j.ijfoodmicro.2019.05.010

[vms31335-bib-0006] Basanisi, M. G. , La Bella, G. , Nobili, G. , Raele, D. A. , Cafiero, M. A. , Coppola, R. , Damato, A. M. , Fraccalvieri, R. , Sottili, R. , & La Salandra, G. (2022). Detection of *Coxiella burnetii* DNA in sheep and goat milk and dairy products by droplet digital PCR in south Italy. International Journal of Food Microbiology, 366, 109583. 35182931 10.1016/j.ijfoodmicro.2022.109583

[vms31335-bib-0007] Borenstein, M. , Hedges, L. V. , Higgins, J. P. T. , & Rothstein, H. R. (2011). Introduction to meta‐analysis. Wiley.

[vms31335-bib-0008] Capuano, F. , Mancusi, A. , Casalinuovo, F. , Perugini, A. , Proroga, Y. , Guarino, A. , & Berri, M. (2012). Real‐time PCR‐based detection of *Coxiella burnetii* in cheeses. European Food Research and Technology, 235, 1181–1186.

[vms31335-bib-0009] Egger, M. , Davey Smith, G. , Schneider, M. , & Minder, C. (1997). Bias in meta‐ analysis detected by a simple, graphical test. BMJ, 315, 629–634. 9310563 10.1136/bmj.315.7109.629 PMC2127453

[vms31335-bib-0010] Eldin, C. , Angelakis, E. , Renvoisé, A. , & Raoult, D. (2013). *Coxiella burnetii* DNA, but not viable bacteria, in dairy products in France. American Journal of Tropical Medicine and Hygiene, 88, 765–769. 23382158 10.4269/ajtmh.12-0212 PMC3617866

[vms31335-bib-0011] Eldin, C. , Mélenotte, C. , Mediannikov, O. , Ghigo, E. , Million, M. , Edouard, S. , Mege, J.‐L. , Maurin, M. , & Raoult, D. (2017). From Q fever to *Coxiella burnetii* infection: A paradigm change. Clinical Microbiology Reviews, 30(1), 115–190. 27856520 10.1128/CMR.00045-16 PMC5217791

[vms31335-bib-0012] Enright, J. B. , Sadler, W. , & Thomas, R C. (1957). Pasteurization of milk containing the organism of Q fever. American Journal of Public Health, 47, 695–700. 13424814 10.2105/ajph.47.6.695 PMC1551060

[vms31335-bib-0030] de Fátima Nascimento, C. , de Mello , V. V. C. , Machado, R. Z. , André, M. R. , & Bürger, K. P. (2021). Molecular detection of *Coxiella burnetii* in unstandardized minas artisanal cheese marketed in Southeastern Brazil. Acta Tropica, 220, 105942. 33951421 10.1016/j.actatropica.2021.105942

[vms31335-bib-0013] Fusco, V. , Chieffi, D. , Fanelli, F. , Logrieco, A. F. , Cho, G. S. , Kabisch, J. , Böhnlein, C. , & Franz, C. M. (2020). Microbial quality and safety of milk and milk products in the 21st century. Comprehensive Reviews in Food Science and Food Safety, 19(4), 2013–2049. 33337106 10.1111/1541-4337.12568

[vms31335-bib-0014] Gale, P. , Kelly, L. , Mearns, R. , Duggan, J. , & Snary, E. L. (2015). Q fever through consumption of unpasteurised milk and milk products – A risk profile and exposure assessment. Journal of Applied Microbiology, 118(5), 1083–1095. 25692216 10.1111/jam.12778

[vms31335-bib-0015] Galiero, A. , Fratini, F. , Cammà, C. , Di Domenico, M. , Curini, V. , Baronti, I. , Turchi, B. , & Cerri, D. (2016). Occurrence of *Coxiella burnetii* in goat and ewe unpasteurized cheeses: Screening and genotyping. International Journal of Food Microbiology, 237, 47–54. 27543815 10.1016/j.ijfoodmicro.2016.08.008

[vms31335-bib-0016] Guatteo, R. , Beaudeau, F. , Joly, A. , Seegers, H. , & Fourichon, C. (2011). Risk factors for *Coxiella burnetii* infection in French dairy goat herds. Veterinary Research, 42(1), 1–9.21314969

[vms31335-bib-0017] Gyuranecz, M. , Denes, B. , Hornok, S. , Kovacs, P. , Horvath, G. , Jurkovich, V. , Varga, T. , Hajtos, I. , Szabo, R. , Magyar, T. , Vass, N. , Hofmann‐Lehmann, R. , Erdélyi, K. , Bhide, M. , & Dán, Á. (2012). Prevalence of *Coxiella burnetii* in Hungary: Screening of dairy cows, sheep, commercial milk samples, and ticks. Vector Borne Zoonotic Dis (Larchmont, NY), 12(8), 650–653. 10.1089/vbz.2011.0953 22651386

[vms31335-bib-0018] Hamidiyan, N. , Salehi‐Abargouei, A. , Rezaei, Z. , Dehghani‐Tafti, R. , & Akrami‐Mohajeri, F. (2018). The prevalence of *Listeria* spp. food contamination in Iran: A systematic review and meta‐analysis. Food Research International, 107, 437–450. 29580505 10.1016/j.foodres.2018.02.038

[vms31335-bib-0019] Hirai, A. , Nakama, A. , Chiba, T. , & Kai, A. (2012). Development of a method for detecting *Coxiella burnetii* in cheese samples. Journal of Veterinary Medical Science, 74(2), 175–180. 21979453 10.1292/jvms.11-0023

[vms31335-bib-0020] Jana, M. , Adriana, V. , & Eva, K. (2020). Evaluation of DNA extraction methods for culture‐independent real‐time PCR‐based detection of *Listeria monocytogenes* in cheese. Food Analytical Methods, 13(3), 667–677.

[vms31335-bib-0021] Kargar, M. , Rashidi, A. , Doosti, A. , Najafi, A. , & Ghorbani‐Dalini, S. (2015). The sensitivity of the PCR method for detection of *Coxiella burnetii* in the milk samples. Zahedan Journal of Research in Medical Sciences, 17(6), e988.

[vms31335-bib-0022] Khanal, B. K. S. , Pradhan, M. , & Bansal, N. (2019). Cheese: Importance and introduction to basic technologies. Journal of Food Science and Technology Nepal, 11, 14–24.

[vms31335-bib-0023] Khaneghah, A. M. , Fakhri, Y. , & Sant'Ana, A. S. (2018). Impact of unit operations during processing of cereal‐based products on the levels of deoxynivalenol, total aflatoxin, ochratoxin A, and zearalenone: A systematic review and meta‐analysis. Food Chemistry, 268, 611–624. 30064804 10.1016/j.foodchem.2018.06.072

[vms31335-bib-0024] Khaneghah, A. M. , Kamani, M. H. , Fakhri, Y. , Coppa, C. F. S. C. , de Oliveira, C. A. F. , & Sant'Ana, A. S. (2019). Changes in masked forms of deoxynivalenol and their co‐occurrence with culmorin in cereal‐based products: A systematic review and meta‐analysis. Food Chemistry, 294, 587–596. 31126504 10.1016/j.foodchem.2019.05.034

[vms31335-bib-0025] Khanzadi, S. , Jamshidi, A. , Razmyar, J. , & Borji, S. (2014). Identification of *Coxiella burnetii* by touch‐down PCR assay in unpasteurized milk and dairy products in North‐East of Iran. Iranian Journal of Veterinary Medicine, 8, 15–19.

[vms31335-bib-0026] Li, X. , Dusseldorp, E. , Su, X. , & Meulman, J. J. (2020). Multiple moderator meta‐analysis using the R‐package Meta‐CART. Behavior Research Methods, 52(6), 2657–2673. 32542441 10.3758/s13428-020-01360-0 PMC7725699

[vms31335-bib-0027] Moher, D. , Liberati, A. , Tetzlaff, J. , Altman, D. G. , & PRISMA Group . (2009). Preferred reporting items for systematic reviews and meta‐analyses: The PRISMA statement. Annals of Internal Medicine, 151(4), 264–269. 19622511 10.7326/0003-4819-151-4-200908180-00135

[vms31335-bib-0028] Mosleh, N. , Moslehishad, M. , & Moosakhani, F. (2018). Detection of *Coxiella burnetii* by real‐time PCR in raw milk and traditional cheese distributed in Tehran province. Journal of Pharmaceutical & Health Sciences, 6(2), 149–155.

[vms31335-bib-0029] Mura, A. , Sanna, G. , Chessa, G. , Piras, D. , & Tola, S. (2014). Occurrence of *Coxiella burnetii* in dairy cattle and farm environment in a rural area of Sardinia (Italy). Annals of Agricultural and Environmental Medicine, 21(2), 247–250.

[vms31335-bib-0031] Owusu‐Kwarteng, J. , Akabanda, F. , Agyei, D. , & Jespersen, L. (2020). Microbial safety of milk production and fermented dairy products in Africa. Microorganisms, 8(5), 752. 32429521 10.3390/microorganisms8050752 PMC7285323

[vms31335-bib-0032] Ozgen, E. K. , Kilicoglu, Y. , Yanmaz, B. , Ozmen, M. , Ulucan, M. , Bagatir, P. S. , Putur, E. K. , Ormanci, S. , Okumus, B. , Yilmaz, S. I. , Karasahin, O. , Aslan, M. H. , Ozturk, M. , Birinci, A. , Bilgin, K. , Cayci, Y. T. , & Tanyel, E. (2022). Molecular epidemiology of *Coxiella burnetii* detected in humans and domestic ruminants in Turkey. Veterinary Microbiology, 273, 109519. 35932517 10.1016/j.vetmic.2022.109519

[vms31335-bib-0033] Pearson, T. , Hornstra, H. M. , Hilsabeck, R. , Gates, L. T. , Olivas, S. M. , Birdsell, D. M. , Hall, C. M. , German, S. , Cook, J. M. , Seymour, M. L. , Priestley, R. A. , Kondas, A. V. , Clark Friedman, C. L. , Price, E. P. , Schupp, J. M. , Liu, C. M. , Price, L. B. , Massung, R. F. , Kersh, G. J. , & Keim, P. (2014). High prevalence and two dominant host‐specific genotypes of *Coxiella burnetii* in U.S. milk. BMC Microbiology, 14, 41. 24533573 10.1186/1471-2180-14-41 PMC3936997

[vms31335-bib-0034] Petruzzelli, A. , Amagliani, G. , Micci, E. , Foglini, M. , Di Renzo, E. , Brandi, G. , & Tonucci, F. (2013). Prevalence assessment of *Coxiella burnetii* and verocytotoxin‐producing Escherichia coli in bovine raw milk through molecular identification. Food Control, 32, 532–536.

[vms31335-bib-0035] Rabaza, A. , Fraga, M. , Corbellini, L. G. , Turner, K. M. , Riet‐Correa, F. , & Eisler, M. C. (2021). Molecular prevalence of *Coxiella burnetii* in bulk‐tank milk from bovine dairy herds: Systematic review and meta‐analysis. One Health, 12, 100208. 33553561 10.1016/j.onehlt.2020.100208 PMC7846927

[vms31335-bib-0036] Rozental, T. , Faria, L. S. D. , Forneas, D. , Guterres, A. , Ribeiro, J. B. , Araújo, F. R. , Lemos, E. R. S. , & Silva, M. R. (2020). First molecular detection of *Coxiella burnetii* in Brazilian artisanal cheese: A neglected food safety hazard in ready‐to‐eat raw‐milk product. Brazilian Journal of Infectious Diseases, 24, 208–212. 10.1016/j.bjid.2020.05.003 PMC9392133 32563680

[vms31335-bib-0037] Sutton, A. J. , Abrams, K. R. , & Jones, D. R. (2001). An illustrated guide to the methods of meta‐analysis. Journal of Evaluation in Clinical Practice, 7(2), 135–148. 11489039 10.1046/j.1365-2753.2001.00281.x

[vms31335-bib-0038] Szymańska‐Czerwińska, M. , Jodełko, A. , Zaręba‐Marchewka, K. , & Niemczuk, K. (2019). Shedding and genetic diversity of *Coxiella burnetii* in Polish dairy cattle. PLoS ONE, 14(1), e0210244. 30629637 10.1371/journal.pone.0210244 PMC6328121

[vms31335-bib-0039] Tan, T. S. E. , Hernandez‐Jover, M. , Hayes, L. M. , Wiethoelter, A. K. , Firestone, S. M. , Stevenson, M. A. , & Heller, J. (2022). Identifying scenarios and risk factors for Q fever outbreaks using qualitative analysis of expert opinion. Zoonoses and Public Health, 69(4), 344–358. 35243790 10.1111/zph.12923 PMC9310758

[vms31335-bib-0040] Taylor, S. C. , Nadeau, K. , Abbasi, M. , Lachance, C. , Nguyen, M. , & Fenrich, J. (2019). The ultimate qPCR experiment: Producing publication quality, reproducible data the first time. Trends in Biotechnology, 37(7), 761–774. 10.1016/j.tibtech.2018.12.002 30654913

[vms31335-bib-0041] Viechtbauer, W. (2010). Conducting meta‐analyses in R with the metafor package. Journal of Statistical Software, 36(3), 1–48.

[vms31335-bib-0042] Valkovska, L. , Mališevs, A. , Kovaļenko, K. , Bērziņš, A. , & Grantiņa‐Ieviņa, L. (2021). DNA in milk, milk products, and fermented dairy products. Journal of Veterinary Research, 65(4), 441–447. 35111997 10.2478/jvetres-2021-0055 PMC8775727

[vms31335-bib-0043] Wang, N. (2018). How to conduct a meta‐analysis of proportions in R: A comprehensive tutorial. New York, NY, John Jay College of Criminal Justice, 0–62.

[vms31335-bib-0044] WOAH . (2018). Q Fever. 3.1.17. Available from: https://www.woah.org/fileadmin/Home/eng/Health_standards/tahm/3.01.17_Q_FEVER.pdf

